# Multi-tissue miRNA networks in preterm birth: from molecular mechanisms to precision medicine

**DOI:** 10.3389/fimmu.2026.1948569

**Published:** 2026-09-22

**Authors:** Pei Zhou, Bin Song, Ping Yan, Shicang Yu, Dan Wang

**Affiliations:** 1 Department of Gynecology and Obstetrics, The First Affiliated Hospital (Southwest Hospital) of Army Medical University, Chongqing, China; 2 Department of Stem Cell and Regenerative Medicine, Institute of Pathology and Southwest Cancer Center, The First Affiliated Hospital (Southwest Hospital) of Army Medical University, Chongqing, China

**Keywords:** biomarker, microRNA, molecular mechanism, multi-tissue integration, preterm birth, tissue specificity

## Abstract

Preterm birth (PTB) remains the leading cause of neonatal mortality and long-term complications worldwide, yet its molecular pathogenesis—particularly the coordinated regulation across different gestational tissues—is poorly understood. MicroRNAs (miRNAs) are key regulators of gene expression and exhibit tissue-specific patterns in pregnancy-related tissues, including the cervix, placenta, and amniotic membrane. This narrative review provides a comprehensive and integrative overview from a perspective of putative cross-tissue miRNA crosstalk. We summarize the differential miRNA expression profiles across gestational tissues and their associations with PTB, the tissue-specific mechanisms by which miRNAs regulate inflammatory responses, extracellular matrix remodeling, apoptosis, and barrier disruption, as well as the synergistic effects of multi-tissue-derived miRNAs and their potential as predictive biomarkers. We also evaluate the current status of miRNA-based therapeutic strategies. Our overview reveals that miRNA mimics (e.g., miR-21-5p, miR-199a-3p) and antagonists hold preclinical therapeutic potential in modulating inflammatory cascades and preserving cervical barrier function. However, clinical translation faces three major bottlenecks: the lack of pregnancy-specific delivery systems, safety concerns arising from the multi-target nature of miRNAs, and the challenge of targeting multi-tissue synergistic pathological changes with single-agent interventions. Emerging strategies—including exosome-based delivery, ligand-modified nanocarriers, and combination therapies—are currently being explored. The multi-tissue miRNA framework proposed in this review integrates miRNA biomarkers for early risk stratification with targeted therapeutic interventions, providing a new theoretical foundation for research directions in personalized risk assessment and targeted intervention strategies for PTB.

## 1 Introduction

PTB, defined as delivery occurring at or after 28 weeks but before 37 weeks of gestation, represents a prevalent obstetric complication affecting approximately 11% of pregnancies worldwide and serves as the predominant contributor to elevated perinatal morbidity and mortality rates (1). Epidemiological surveys indicate that roughly 15 million preterm infants are delivered globally each year, with over 1 million neonatal deaths attributable to this condition, establishing preterm birth as a critical public health challenge demanding urgent resolution (2, 3). Although clinical investigations have identified numerous risk factors, approximately 50% of cases remain etiologically unexplained, underscoring the pathological complexity of this condition (4, 5). Emerging research has illuminated the pivotal role of epigenetic regulatory mechanisms, particularly miRNAs, in pregnancy maintenance and parturition initiation (6). miRNAs constitute a class of small non-coding RNAs approximately 22 nucleotides in length that achieve precise gene expression modulation through specific binding to the 3’ untranslated regions (UTRs) of target mRNAs (7). Accumulating evidence demonstrates that miRNAs participate in regulating multiple pregnancy-related physiological processes, including placental development, maternal-fetal immune tolerance, uterine contractility, and cervical remodeling (8–11). Notably, cervical tissue exhibits markedly distinct miRNA expression profiles compared with fetal appendage tissues such as placenta and amniotic membrane(AM), suggesting that these tissue-specific miRNAs may form a dynamic interorgan signaling landscape, which is hypothesized to collectively contribute to the pathogenesis of preterm birth (12, 13). Nevertheless, current investigations remain largely confined to analyses of isolated tissues or singular mechanisms, lacking systemic comparative approaches. This review integrates and analyzes miRNA expression signatures from a perspective of putative cross-tissue miRNA crosstalk, elucidating both shared and distinct features along with their synergistic effects. Our overview not only provides novel insights into the complex etiology of preterm birth but also establishes a theoretical foundation for developing tissue-specific diagnostic biomarkers and precision therapeutic targets.

## 2 Classification and etiology of preterm birth

### 2.1 Classification of preterm birth

Preterm birth is defined as delivery occurring between 28 completed weeks and less than 37 weeks of gestation (i.e., <259 days). According to criteria established by the World Health Organization (WHO), preterm birth is strictly characterized as the termination of pregnancy prior to 37 gestational weeks (14). Based on stratification by gestational age, preterm birth can be subclassified into the following categories: extremely preterm (≤28 weeks), very preterm (28^+1–^32^0^ weeks), moderately preterm (32^+1–^34^0^ weeks), and late preterm (34^+1^–36^+6^ weeks) (15, 16). Clinically, preterm birth manifests as two distinct phenotypes: spontaneous preterm birth (sPTB) and iatrogenic preterm birth (IPTB). sPTB refers to unplanned delivery before 37 weeks of gestation, encompassing both preterm labor with intact membranes and preterm premature rupture of membranes (PPROM). Its precise etiopathogenesis remains incompletely understood. In contrast, iatrogenic preterm birth results from medically indicated, planned delivery due to maternal or fetal complications—though it should be noted that not all such deliveries are unequivocally medically justified (17–20). Epidemiological studies indicate that sPTB accounts for approximately 40–45% of all preterm births, with 25–30% attributed to PPROM and 30–35% to medically indicated deliveries (21–23). Recent investigations have demonstrated that singleton pregnancies conceived through assisted reproductive technologies (ART), including *in vitro* fertilization (IVF) or intracytoplasmic sperm injection (ICSI), exhibit significantly elevated risks of both spontaneous and iatrogenic preterm birth compared with naturally conceived pregnancies (24, 25). Furthermore, a history of prior sPTB has been established as a major risk factor for recurrent preterm delivery (26). Given the profound adverse consequences of preterm birth on maternal and neonatal health, the identification of early biomarkers for accurate risk prediction before clinical onset holds substantial implications for preventive strategies and clinical management. This approach also opens avenues for targeted intervention strategies to optimize pregnancy outcomes.

### 2.2 Research advances in miRNA regulatory network-based biomarkers for preterm birth prediction

PTB is a multifactorial outcome influenced by diverse determinants. Established risk factors fall into two principal categories: 1) Maternal baseline characteristics: Extreme age (<17 or >40 years) (27), low educational attainment (28), short interpregnancy interval (29), and preconception low body mass index (BMI) <18.5 kg/m² (30). 2) Pregnancy-associated pathological factors: History of cervical surgery (31), repeated uterine curettage (32), multifetal gestation (33), and cervical shortening or abnormal angulation (34). Although epidemiological associations exist between these factors and preterm birth risk, their predictive utility is constrained by population heterogeneity and complex gene-environment interactions, impeding precise individualized risk assessment. Recent research has focused on identifying precise biomarkers. While current biomarkers have achieved modest progress in preterm birth prediction, their translational application faces notable limitations. Fetal fibronectin (fFN) testing, despite its well-defined threshold discrimination capability (positive predictive value of 37–67% for preterm birth before 34 weeks at >200 ng/mL), suffers from critical drawbacks: its predictive performance deteriorates sharply in the intermediate-risk range (10–200 ng/mL, negative predictive value only 75–85%), and it fails to distinguish between spontaneous and infection-associated preterm birth subtypes (35). Plasma cell-free RNA profiling, though revealing preterm birth-associated pathways (e.g., collagen metabolism via COL4A2/COL5A1) through transcriptome-wide analysis, confronts three major challenges: 1) prohibitive costs (15- to 20-fold higher than conventional ELISA); 2) stringent requirement for gestational age-matched reference intervals (necessitating independent modeling per 2-week window); and 3) extreme sensitivity to preanalytical variables (e.g., blood collection tubes, centrifugation protocols), collectively hindering routine clinical implementation (36). Inflammation markers (e.g., IL-6/IL-8) and HSPA5 protein, despite mechanistic links to chorioamnionitis, exhibit inherent shortcomings as systemic indicators: 1) poor tissue specificity (elevated serum IL-6 may originate from non-genital tract inflammation like gingivitis); 2) narrow dynamic range (physiological fluctuations during pregnancy can reach 50–70% of pathological levels); and 3) biomarker overlap with complications like preeclampsia (e.g., HSPA5 dysregulation occurs in both conditions), severely compromising diagnostic specificity (37).

In contrast to the traditional biomarkers described above, miRNA regulatory networks have the potential to overcome these limitations: 1) Multitarget cooperativity—single miRNAs (e.g., miR-150-5p) can simultaneously regulate hundreds of mRNAs, suppressing both inflammatory and fibrotic pathways, enabling comprehensive detection of pathway dysregulation (38); 2) Gestational stage-specific dynamics—longitudinal studies demonstrate that exosomal miRNA profiles sensitively track pregnancy progression, exemplified by miR-515-5p exhibiting temporally correlated expression with cervical remodeling from mid-to-late gestation (39); 3) Mechanism-guided preclinical therapeutic potential—experimentally validated axes (e.g., miR-223-3p/IL-6R) reveal direct intervention nodes, where miRNA binding to the 3’-UTR of IL-6R mRNA suppresses its expression, attenuating downstream NF-κB/STAT3 activation (40). Recent studies identified placenta-/maternal blood-derived exosomal miRNAs (e.g., miR-515-5p, miR-518b) as key regulators of inflammation or placental dysfunction in preterm birth, with certain combinatorial panels achieving promising prediction AUCs >0.85 in discovery-phase cohorts, which cannot yet outperform conventional clinical markers (41).

Moving forward, integrating miRNA networks with clinical data to construct dynamic prediction models will be essential for personalized risk assessment and targeted intervention strategies interventions in PTB.

## 3 Structure and function of miRNAs

### 3.1 Biological characteristics of miRNAs and the intrinsic nature of their regulatory networks

miRNAs are highly conserved 18–25 nucleotide non-coding RNAs that mediate post-transcriptional gene silencing by binding to complementary sites, primarily within the 3′ untranslated regions (3′UTRs) of target mRNAs, leading to mRNA degradation and/or translational repression (42–44). As core regulators of gene expression networks, miRNAs govern embryonic development, cell-cycle progression and other fundamental biological processes, and their dysregulation is associated with multiple pathological conditions (45–48). Since the landmark discovery of the temporal regulator lin-4 in C. elegans in 1993 (49) and subsequent identification of the human let-7 family (50), research on miRNAs has underscored their remarkable evolutionary conservation. The current human miRNA repertoire comprises 2,654 annotated species (miRBase v22) (51, 52), predicted to regulate over half of all protein-coding genes through an intricate regulatory architecture: individual miRNAs typically target hundreds of mRNAs, while single mRNAs often undergo combinatorial regulation by multiple miRNAs (53, 54). This network-level functionality positions miRNAs as master regulators of cellular homeostasis. Pathological disruptions in the processing machinery from pri-miRNA to mature miRNA have been conclusively linked to cancer, neurodegenerative disorders, and other diseases (55–57).

### 3.2 Pathways of miRNA biogenesis

The maturation process of miRNAs in mammals involves three key molecular forms: primary transcripts (pri-miRNAs), precursors (pre-miRNAs), and mature miRNAs (58). Their biosynthesis is accomplished through two main pathways (**Figure 1**): the canonical nuclear pathway and non-canonical pathways (59). In the canonical nuclear pathway, pri-miRNAs are transcribed by RNA polymerase II (Pol II) or III (Pol III) (60). Intron-derived miRNAs are generally transcribed by Pol II according to their genomic localization (51, 61), while polymerase type does not alter miRNA final function (61–63). The initial pri-miRNA transcript contains the mature miRNA sequence within its stem-loop structure (64). The Drosha–DGCR8 complex recognizes structural motifs on pri-miRNAs (65–67), and cleaves them into pre-miRNAs (68–71). These pre-miRNAs are then exported to the cytoplasm via the Exportin-5/RAN-GTP system (72–75). In the cytoplasm, Dicer (assisted by TRBP) cleaves pre-miRNAs into a miRNA/miRNA* duplex (76–79). The guide strand is loaded onto AGO proteins to form RISC, while the passenger strand is degraded (80–84).

**Figure 1:**
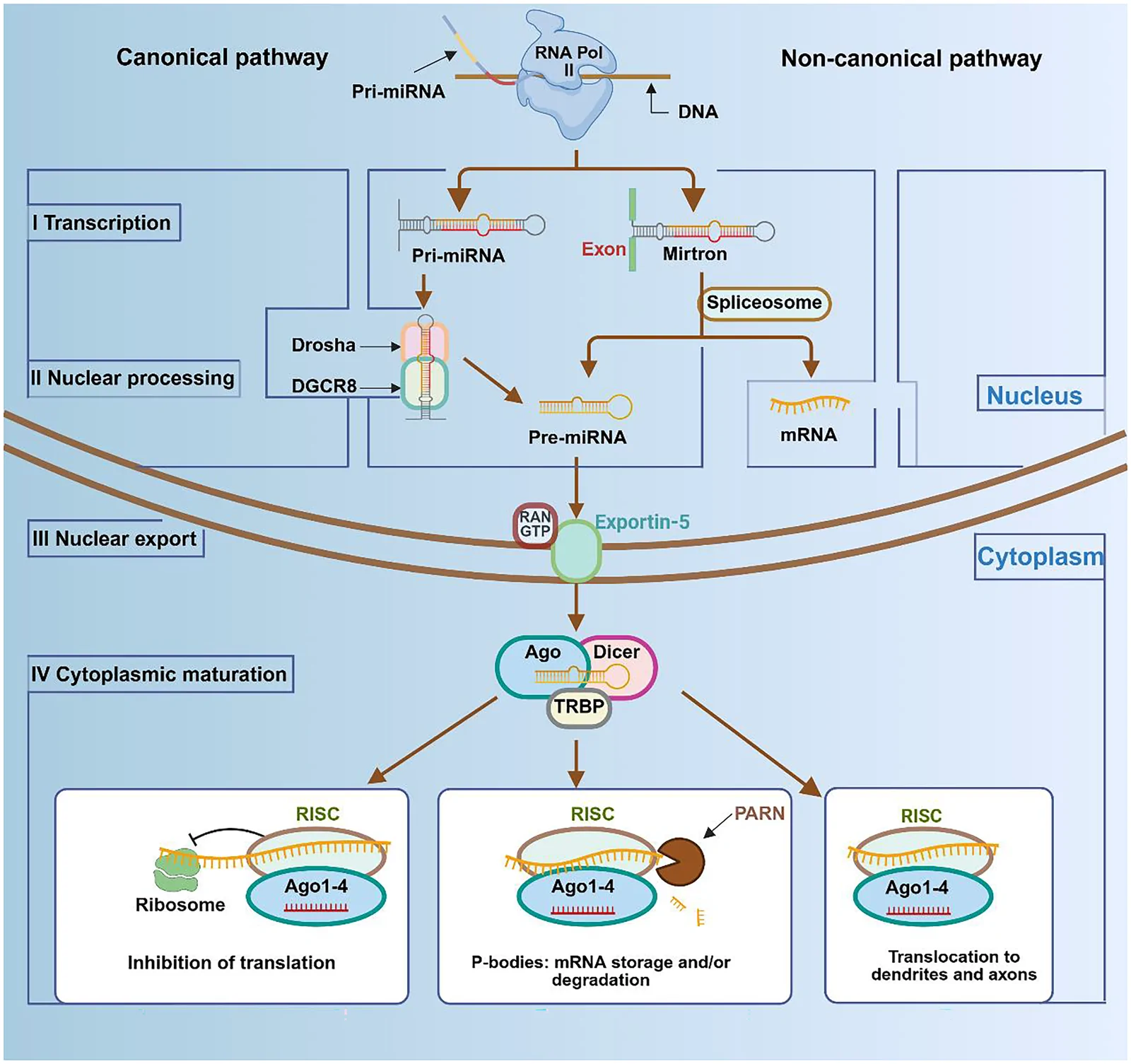
Biogenesis of microRNAs in mammals: canonical and mirtron pathways. Left: Canonical pathway. RNA polymerase II/III transcribes miRNA genes into primary miRNAs (pri-miRNAs) with a 5’ cap and 3’ polyA tail. The Drosha-DGCR8 complex cleaves pri-miRNA into pre-miRNA, which is exported to the cytoplasm by Exportin-5/RAN-GTP. Dicer (with TRBP) cleaves pre-miRNA into a miRNA/miRNA* duplex. The guide strand is loaded into Argonaute (AGO) to form the RNA-induced silencing complex (RISC). Right: Mirtron pathway. Intron-derived pre-mRNA forms a lariat after splicing. The debranching enzyme linearizes it into a pre-miRNA-like hairpin, which is exported by Exportin-5 and cleaved by Dicer for RISC assembly.

In the non-canonical mirtron pathway, intron-derived miRNAs are processed by the spliceosome into lariats, debranched, folded into pre-miRNA-like molecules, exported by Exportin-5, and matured via the Dicer-mediated pathway (85–87) (**Figure 1**).

### 3.3 Roles of miRNAs in reproductive disorders

Given their broad gene regulatory capacity, miRNAs have been widely investigated in multiple reproductive disorders. In recurrent pregnancy loss (RPL), dysregulated miRNAs such as miR-146a-5p and miR-155-5p have been linked to impaired immune tolerance at the maternal–fetal interface and aberrant TLR–NLR signaling pathways that disrupt uterine homeostasis (88, 89). In preeclampsia (PE)—a condition sharing inflammatory and angiogenic dysregulation with preterm birth—the placenta-specific C19MC cluster and miR-210 are consistently reported as dysregulated, contributing to aberrant trophoblast invasion, endothelial dysfunction, and elevated maternal circulating levels of anti-angiogenic factors (90). In fetal growth restriction (FGR), altered expression of miR-16-5p, miR-590-3p, and miR-206 has been associated with impaired placental angiogenesis through targeting of VEGF, PIGF, and MMP9, critical regulators of trophoblast invasion and nutrient transport (91). In the context of infertility, miRNAs embedded in extracellular vesicles have been shown to regulate gametogenesis, embryo implantation, and endometrial receptivity, with specific signatures in follicular fluid and seminal plasma emerging as potential diagnostic indicators (92, 93). These examples underscore a common theme: miRNAs serve as critical nodes connecting inflammation, immune regulation, and tissue remodeling across diverse obstetric and gynaecological syndromes.

In the context of PTB, however, a unique dimension is added by the involvement of multiple gestational tissues—cervix, placenta, amniotic membrane, and maternal circulation—each contributing distinct miRNA signatures that collectively orchestrate the pathological cascade. This multi-tissue interplay, rather than isolated alterations in a single compartment, forms the central rationale for adopting a network-based perspective in this review. Accordingly, the following sections focus on the crosstalk between miRNAs and inflammatory signaling across these four tissue compartments and their pathological roles in PTB.

## 4 Tissue-specific miRNAs and preterm birth

### 4.1 Cervical miRNA regulatory signatures and the mechanisms of preterm birth initiation

Cervical ripening is a critical step in the initiation of parturition, and its premature activation can lead to preterm birth. In recent years, multiple studies have revealed that aberrant expression of miRNAs in cervical tissue is closely associated with the occurrence of preterm birth (94).

#### 4.1.1 Characteristics of the cervical miRNA expression profile

Burris et al. conducted a prospective cohort study of cervical miRNA expression profiles in 25 women with preterm birth and 49 women with term delivery (at 17.1 ± 4.8 weeks of gestation). They found that the overall expression of 346 miRNAs was significantly elevated in the preterm birth group, with particularly marked upregulation of miR-143-3p, miR-30e-3p, and miR-199b-3p. Enrichment analysis suggested that these differentially expressed miRNAs are primarily involved in the regulation of inflammatory cell signal transduction (95). This finding indicates that aberrant cervical miRNA expression may precede clinical symptoms and thus holds early warning value.

#### 4.1.2 Anti-inflammatory protective role of miR-199a-3p

Peng et al., through analysis of clinical samples (52 preterm birth, 60 PPROM, and 70 term delivery cases), found that miR-199a-3p expression was significantly reduced in cervical epithelial tissues of patients in the preterm labor group and the PPROM group. Mechanistic studies demonstrated that miR-199a-3p directly targets the 3′UTR of HMGB1. Overexpression of miR-199a-3p reduced HMGB1 protein levels, attenuated downstream TLR4/NF-κB pathway activation, and decreased the release of inflammatory cytokines (IL-6, TNF-α). In a lipopolysaccharide (LPS)-induced preterm birth mouse model, *in vivo* transfection with miR-199a-3p mimics significantly suppressed cervical inflammation (96). These results reveal a protective role of the miR-199a-3p/HMGB1 axis in maintaining cervical homeostasis.

#### 4.1.3 Disruption of cervical barrier function by miR-143-3p/miR-145-5p

Anton et al. overexpressed miR-143-3p and miR-145-5p in cervical epithelial cells and observed increased cell permeability, enhanced apoptosis, downregulated BCL2 expression, and cell cycle arrest, suggesting that these two miRNAs disrupt cervical epithelial barrier function through multiple mechanisms (97). Further studies revealed that sterile supernatants from Gardnerella vaginalis and Lactobacillus iners, both closely associated with preterm birth, upregulated miR-143-3p and miR-145-5p expression and increased cell permeability, whereas Lactobacillus crispatus did not exert such effects (98). Dude et al. found that culture supernatants from Mobiluncus mulieris induced the upregulation of preterm birth-associated miRNAs including miR-21-5p, miR-146a-5p, miR-143-3p, and miR-494-3p in cervical epithelial cells (99). Collectively, these findings highlight the important role of the cervical-vaginal microbiota–miRNA interaction axis in the pathogenesis of preterm birth.

### 4.2 The hub role of the placental miRNA interactions in preterm birth

The placenta is the core functional organ of the maternal–fetal interface. Aberrant miRNA expression in the placenta can lead to placental dysfunction and thereby contribute to the pathogenesis of preterm birth (100–102).

#### 4.2.1 Regulation of placental miR-21-5p by environmental exposure

Ran et al., using a birth cohort study, mouse models, and cellular experiments, demonstrated that ambient PM2.5 exposure is positively correlated with preterm birth risk. The underlying mechanism involves downregulation of placental miR-21-5p expression, which activates the TLR4/NF-κB/NLRP3 inflammatory axis. Administration of miR-21-5p mimics or an NF-κB inhibitor reversed this effect (103). This study establishes the miR-21-5p/NF-κB axis as a central mediator in environmentally induced preterm birth.

#### 4.2.2 Immune-regulatory miRNAs in infection-associated preterm birth

Bhati et al. identified significantly upregulated serum levels of miR-223-3p and miR-150-5p in patients with sPTB complicated by *Chlamydia trachomatis* or *Mycoplasma* infection. These miRNAs showed negative correlations with placental inflammatory markers (IL-6ST/IL-6/MMP-14) and positive correlations with TGF-β signaling pathway-related genes, suggesting that these two miRNAs exert predominantly anti-inflammatory immunomodulatory effects in infection-associated preterm birth (104).

#### 4.2.3 Differential regulation of the C19MC miRNA family

Hromadnikova et al. found that the C19MC miRNA family (including miR-515-5p, miR-518b, and others) was upregulated in pregnancies with PTB but downregulated in those with preterm premature rupture of membranes (PPROM). Moreover, in the PTB group, miR-515-5p levels showed a strong positive correlation with white blood cell count, indicating a differential regulatory role of the C19MC miRNA family across preterm birth subtypes (90).

#### 4.2.4 Inflammatory transcriptional signatures in placental villi during intra-amniotic infection

Ackerman et al. performed RNA sequencing to compare preterm birth cases with intra-amniotic infection complicated by chorioamnionitis versus idiopathic preterm birth. They found that miR-133a-3p and miR-223-3p were significantly upregulated in placental villous tissues of the infection group, with enrichment in acute inflammatory pathways, whereas the transcriptional response in the basal decidual tissue was comparatively weaker (105).

### 4.3 Circulating miRNA communication: systemic regulation at the maternal–fetal interface

Maternal circulating miRNAs offer the advantage of convenient sampling and have become a focus of biomarker research in preterm birth (106, 107).

#### 4.3.1 Plasma miRNAs as predictive biomarkers for preterm birth

Ramzan et al. found that plasma levels of miR-451a, miR-223-3p, let-7a-5p, and miR-126-3p were significantly downregulated in pregnant women with sPTB. Pathway analysis revealed that these miRNAs are closely associated with inflammation, apoptosis, and mitochondrial biogenesis (108). Mavreli et al. reported a significant downregulation of miR-125a-3p in the sPTB group (109).

#### 4.3.2 Role of exosomal miRNAs in maternal–fetal communication

Truong et al. demonstrated that in exosomes derived from extravillous trophoblast cells cultured under hypoxic conditions, the expression of miRNAs promoting spiral artery remodeling was decreased, whereas the expression of miRNAs inhibiting angiogenesis was increased. Plasma levels of miR-517a-3p and miR-517b-3p were significantly elevated in pregnant women with sPTB, suggesting that hypoxia may influence maternal vascular function through exosomal miRNAs (110).

#### 4.3.3 Postpartum miRNA expression and long-term risk

Hromadnikova et al. found that the expression levels of 25 miRNAs in peripheral blood leukocytes of women following preterm birth or PPROM were negatively correlated with neonatal gestational age and birth weight; earlier delivery and lower birth weight were associated with higher miRNA expression levels. Aberrant expression of these miRNAs may reveal the molecular mechanisms underlying increased long-term cardiovascular risk in women who have experienced preterm birth (111).

### 4.4 The amniotic membrane/amniotic fluid miRNA–inflammation feedback interactions and infection-induced preterm birth

Intra-amniotic infection is closely associated with preterm birth, and amniotic fluid miRNAs may serve as non-invasive biomarkers of intrauterine infection.

#### 4.4.1 Role of the miR-548/HMGB1 axis in chorioamnionitis

Son et al. found that in amniotic membrane tissues of patients with preterm birth complicated by acute chorioamnionitis, members of the miR-548 family were significantly reduced, whereas HMGB1 expression was markedly upregulated. Overexpression of the miR-548 family/cluster members *in vitro* suppressed LPS-induced HMGB1 expression and secretion, suggesting that the miR-548/HMGB1 axis represents a potential therapeutic target for this condition (112).

#### 4.4.2 Amniotic fluid miRNAs as predictive biomarkers for adverse neonatal outcomes

Yoshikawa et al. found that levels of miR-4535 and miR-1915-5p were significantly elevated in amniotic fluid of patients with severe chorioamnionitis, and their predictive performance for adverse neonatal outcomes, as assessed by the area under the curve (AUC), was superior to that of 16S rDNA and IL-6. This study also found that prolonged rupture of membranes was associated with further elevation of miRNA expression levels and increased severity of infection (113) (**Table 1** and **Figures 2**, **3**).

**Table 1:** Summary of available studies associated with miRNA function in PTB.

Study	Sample size	Study design	PTB subtype	Gestational age	Objects	Tissue/cells	miRNA	Expression	Pathway/function	Independent validation	Level of evidence	Biomarker status
Burris (95)	25 PTB/49 term	Prospective cohort	sPTB	17.1 ± 4.8 weeks	Human	Cervical	miR-143-3p, miR-30e-3p, miR-199b-3p	Up	Potential inflammatory role	No	observational (human)	Discovery-phase biomarker
Peng (96)	52 PTB/60 PPROM/70 term	Case-control + animal model	PTB	<37 weeks	Human & Mice	Peripheral blood, Cervical epithelial	miR-199a-3p	Down	Pro-inflammatory (HMGB1, TLR4, IL-1β, TNF-α↑)	Yes (*in vivo* mouse model)	observational (human); mechanistic (*in vitro*); animal (*in vivo*)	Discovery-phase biomarker
Anton (97)	Not specified	*In vitro* experimental	PTB	Not specified	Human	Ectocervical, Endocervical	miR-143-3p, miR-145-5p	Up	Promote Apoptosis (BCL2↓), Cell permeability↑, cell cycle arrest	No	observational (human); mechanistic (*in vitro*)	Discovery-phase biomarker
Anton (98)	Not specified	*In vitro* experimental	PTB	Not specified	Human	Ectocervical, Endocervical	miR-143-3p, miR-145-5p, miR-193b-3p, miR-146a-5p, miR-223-3p, miR-148b-3p, miR-15a-5p	Up	Pro-inflammatory (IL-10, IL-6, IL-8↑)	No	observational (human); mechanistic (*in vitro*)	Discovery-phase biomarker
Dude (99)	Not specified	*In vitro* experimental	sPTB	Not specified	Human	Ectocervical	miR-193b-3p, miR-21-5p, miR-146a-5p, miR-148b-3p, miR-223-3p, miR-143-3p, miR-494-3p	Up	Pro-inflammatory (IL-6, IL-8↑)	No	observational (human); mechanistic (*in vitro*)	Discovery-phase biomarker
Ran (103)	Birth cohort + animal model	Prospective cohort + animal model	PTB	30.53 weeks	Human & Mice	Placental, HTR-8/SVneo cells	miR-21-5p	Down	Pro-inflammatory (TLR4,NF-κB, NLRP3↑)	Yes (*in vivo* mouse model)	observational (human); mechanistic (*in vitro*); animal (*in vivo*)	Discovery-phase biomarker
Bhati (104)	sPTB with infection vs. term	Case-control	sPTB	<37 weeks	Human	Non-heparinized blood, Placental	miR-223-3p, miR-150-5p	Up	Anti-inflammatory (IL-6/MMP-14↓; TGF-β↑)	No	observational (human); mechanistic (*in vitro*)	Discovery-phase biomarker
Hromadnikova (90)	PTB/PPROM vs. controls	Case-control	PTB	25^+1^–37weeks	Human	Placental	C19MC cluster (miR-515-5p, miR-519a and other members.)	PTB↑, PPROM↓	PTB: pro-inflammatory (WBC correlation↑); PPROM: distinct pathways	No	observational (human)	Discovery-phase biomarker
Ackerman (105)	IAI-PTB vs. idiopathic PTB	Case-control	PTB(IAI)	25–31 weeks	Human	Placental villous trophoblast, Decidua	miR-133a-3p, miR-223-3p	Up	Pro-inflammatory	No	observational (human)	Discovery-phase biomarker
Ramzan (108)	sPTB vs. term	Case-control	sPTB	28–36 weeks	Human	Plasma	miR-451a, miR-223-3p, let-7a-5p, miR-126-3p	Down	Associated with inflammatory, apoptosis, mitochondrial biosynthesis	No	observational (human)	Discovery-phase biomarker
Mavreli (109)	sPTB vs. term	Nested case-control	sPTB	32^+0^–36^+6^ weeks	Human	Plasma	miR-23b-5p, miR-125a-3p	Down	T cell receptor signaling pathway	No	observational (human)	Discovery-phase biomarker
Truong (110)	sPTB vs. term	Case-control + *in vitro*	sPTB	Before 20 weeks	Human	Plasma	miR-517a-3p, miR-517b-3p	Up	Hypoxia-induced anti-angiogenic miRNAs ↑→ vascular dysfunction	No	observational (human); mechanistic (*in vitro*)	Discovery-phase biomarker
Hromadnikova (111)	PTB/PPROM vs. controls	Case-control	PTB	<37 weeks	Human	Peripheral blood	miR-143-3p, miR-199a-5p	Up	Pro-inflammatory (CRP↑)	No	observational (human)	Discovery-phase biomarker
Son (112)	PTB with chorioamnionitis vs. without	Case-control + *in vitro*	PTB(IAI)	<37 weeks	Human	Amniotic membrane	miR-548 family/cluster members	Down	Anti-inflammatory (HMGB1↓)	No	observational (human); mechanistic (*in vitro*)	Discovery-phase biomarker
Yoshikawa (113)	Severe CAM vs. mild CAM	Case-control	PTB(IAI)	24–37 weeks	Human	Amniotic fluid	miR-4535, miR-1915-5p	Up	Predicts neonatal sepsis (AUC > IL-6/16S rDNA); correlates with infection severity(small sample size; no external validation; risk of overfitting)	No (small sample size; no external validation)	observational (human)	Discovery-phase biomarker

**Figure 2:**
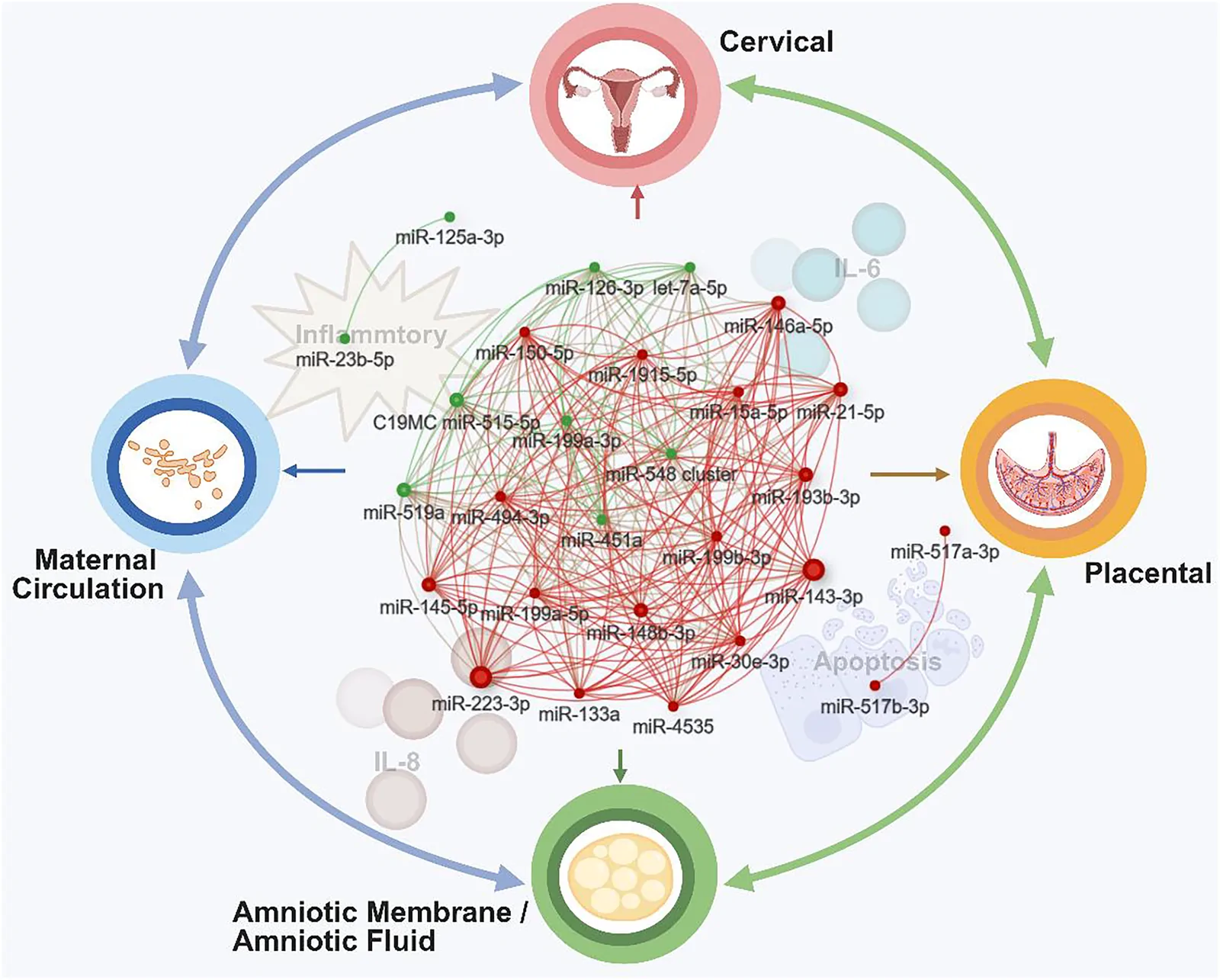
Putative spatial interaction landscape of miRNAs in preterm birth. The miRNA interaction network was visualized using R language with the visNetwork package. The workflow consisted of three main steps. First, data preprocessing was performed: miRNA names were cleaned, composite entries were split, and miRNAs were categorized as up- or down-regulated based on expression keywords. Biological functions were grouped into six categories according to pathway-related keywords (e.g., inflam, apop). Second, network elements were defined: nodes represent miRNAs, with node size proportional to the square root of occurrence frequency; node color indicates expression status (green for up-regulated, red for down-regulated). Edges were established between miRNA pairs sharing the same functional category. Edge color was mixed from the two connected nodes, with transparency set to 70% for miRNAs with identical expression status and 40% for those with divergent status. Edges belonging to the “other” category were hidden to highlight core associations. The edges represent hypothesized associations derived from literature co-occurrence rather than experimentally validated direct regulatory interactions. Finally, dynamic network visualization was generated with arc connections (curvature = 0.3) and force-directed layout (repulsion = -250). Interactive functions including hover-highlighting and expression-status filtering, together with optimized legends, were implemented to display the cooperative regulatory landscape of miRNAs in key pathways such as inflammation and apoptosis.

**Figure 3:**
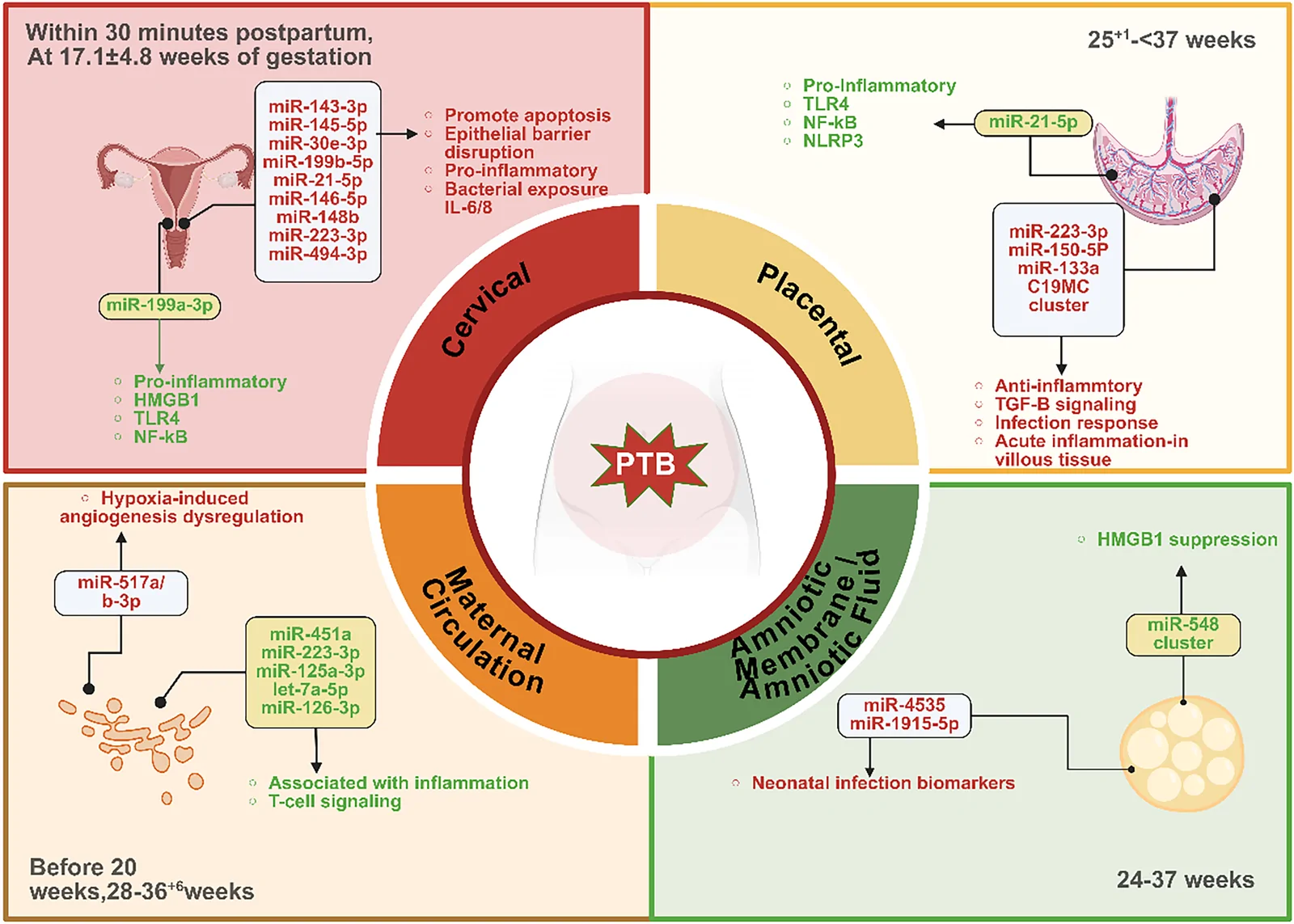
Spatiotemporal dynamics of miRNA regulation in preterm birth. The concentric circle diagram illustrates the putative multi-tissue miRNA signaling landscape associated with PTB. The core represents PTB pathogenesis, radiating outward to four tissue compartments: cervix, placenta, amniotic membrane/amniotic fluid, and circulation. Each tissue layer shows differentially expressed miRNAs (red: upregulation, green: downregulation), with arrows indicating their targeted pathways. Gestational ages at which miRNA expression was detected are annotated for each tissue, revealing the temporal order of miRNA dysregulation across different tissues during PTB progression.

## 5 Current status, bottlenecks, and challenges of miRNA-based targeted therapeutic strategies

### 5.1 miRNA-based therapeutic agents for preterm birth

Although studies directly investigating miRNA-based interventions targeting the pathological processes of preterm birth remain limited in number, several candidate agents have demonstrated feasibility in improving preterm birth outcomes in animal models (114–116). It is important to note that most of these studies are at the stage of tissue-level miRNA modulation or functional validation, and true tissue-specific targeted delivery has not yet been achieved. Based on the tissue compartments, these studies can be broadly categorized into several directions: regulation of uterine contraction, inhibition of placental inflammation, preservation of cervical barrier function, strategies directed toward the amniotic membrane/amniotic fluid, and exosome/stem cell-mediated miRNA delivery (96–98).

#### 5.1.1 miRNA-based strategies directed toward the myometrium

Aberrant uterine contraction is the direct cause of preterm birth, and the myometrium is the effector tissue of contraction. miRNA-based therapeutic strategies targeting uterine smooth muscle represent one of the best-evidenced directions. Deng et al., through transcriptome sequencing of human myometrium during labor, identified miR-206-5p as the most differentially expressed miRNA associated with uterine contraction, with significant downregulation in the labor group. Mechanistic studies revealed that miR-206-5p inhibits gap junction formation and reduces intercellular communication among uterine smooth muscle cells by directly targeting GJA1. In a RU486-induced preterm birth mouse model, tail vein injection of miR-206-5p agomir reduced the preterm birth rate to 43%, with some mice delivering at term, and neonatal survival rates were not significantly different from those in the control group (114). Ying et al. reported that miR-203-3p expression gradually decreases during pregnancy and negatively regulates uterine smooth muscle contraction by targeting the TRPV4 channel. Overexpression of miR-203-3p significantly prolonged gestational duration in an inflammatory preterm birth mouse model, providing a novel strategy for preterm birth prevention and treatment (117). Wang et al. uncovered a novel mechanism of placenta–uterus crosstalk: trophoblast-derived small extracellular vesicles can transport miR-25-3p to uterine smooth muscle, where it regulates Ca²^+^ oscillations and cell contraction by targeting Cav3.2 and SERCA2a, and dysregulation of this pathway is closely associated with preterm birth (118). These studies indicate that multiple miRNA nodes regulating contractile function exist within the myometrium and provide important experimental evidence for the development of miRNA-based preterm birth intervention strategies.

#### 5.1.2 miRNA-based strategies directed toward the placenta

Placental inflammation is a core driver of infection-induced preterm birth, and miRNAs play critical roles in regulating placental inflammatory responses. The miR-21-5p/NF-κB signaling axis is among the most intensively investigated targets. Duan et al. found that in an LPS-induced infection-associated preterm birth mouse model, miR-21-5p expression was significantly downregulated in placental tissue, with NF-κB identified as a direct target gene. Intraperitoneal injection of miR-21-5p mimic restored miR-21-5p expression and suppressed NF-κB as well as downstream inflammatory cytokines IL-6 and TNF-α (116). Of note, the role of miR-21-5p in preterm birth is tissue-specific—it is downregulated in the placenta where it exerts a protective effect, whereas it plays a distinct role in models of white matter injury in preterm infants (119). A systematic review by Guo et al. explicitly states that miRNA-based mimics or inhibitors hold preclinical therapeutic potential for restoring normal placental function, but optimization of placenta-specific delivery systems remains a major challenge (120). Jin et al. also noted that a deeper understanding of miRNA expression and function in human pregnancy, along with the development of novel miRNA-targeting drugs, represents a potentially effective preclinical approach for future intervention of pregnancy-associated diseases, including preterm birth (121).

#### 5.1.3 miRNA-based strategies directed toward the cervix

Cervical ripening and maintenance of barrier function are critical for pregnancy maintenance. To date, several candidate miRNAs have been identified as potential targets for cervical-directed therapy. Peng et al. found that miR-199a-3p expression is significantly reduced in the cervical epithelium of patients with preterm birth, and overexpression of miR-199a-3p inhibits HMGB1 in a targeted manner, blocking the TLR4/NF-κB inflammatory cascade. In an LPS-induced preterm birth mouse model, *in vivo* transfection with miR-199a-3p mimics significantly suppressed cervical inflammation and reduced IL-1β and TNF-α levels, providing *in vivo* evidence of efficacy for cervical-targeted therapy (96). Furthermore, cervical miRNA expression profiling studies have identified multiple potential targets. A systematic review indicated that cervical miRNA expression profiling at 17 weeks of gestation can identify women at high risk for preterm birth, with miR-143-3p, miR-30e-3p, and miR-199b-3p significantly upregulated in the preterm birth group and primarily involved in the regulation of inflammatory signaling pathways (122). Alterations in the cervical–vaginal microbiota can also participate in preterm birth pathogenesis by regulating the expression of miR-143-3p, miR-145-5p, and others (97, 98), providing a new avenue for targeting the microbe–miRNA interaction axis. These findings suggest that local regulation of cervical miRNAs represents a promising direction for future preterm birth prevention. However, current research remains at an early stage, and key issues such as delivery efficiency and long-term safety require further exploration.

#### 5.1.4 miRNA-based strategies directed toward the amniotic membrane/amniotic fluid

Preterm premature rupture of membranes accounts for approximately 25–30% of sPTB, and effective targeted interventions are currently lacking. To date, the only miRNA validated in animal models for amniotic membrane targeting is miR-144-3p. Studies have shown that miR-144-3p suppresses prostaglandin E2 synthesis by inhibiting c-fos and COX2 expression, thereby maintaining uterine quiescence during pregnancy. Overexpression of miR-144-3p in amniotic membrane tissue significantly reduced inflammation-induced PGE2 secretion, suggesting that miR-144-3p mimic has the potential to prevent preterm birth (123). Candidate targets validated *in vitro*: Wang et al. found that miR-150-5p regulates chorionic cell migration and invasion by targeting ADAM19; Son et al. reported that the miR-548 family inhibits HMGB1-mediated inflammatory responses. These targets are currently still in the *in vitro* validation stage and require further evaluation of their therapeutic value in animal models. No studies on miRNA delivery systems specifically targeting the amniotic membrane have been reported to date. Achieving selective targeting of amniotic membrane tissue by miRNA formulations represents the core bottleneck for clinical translation in this field (**Figure 4** and **Table 2**).

**Figure 4:**
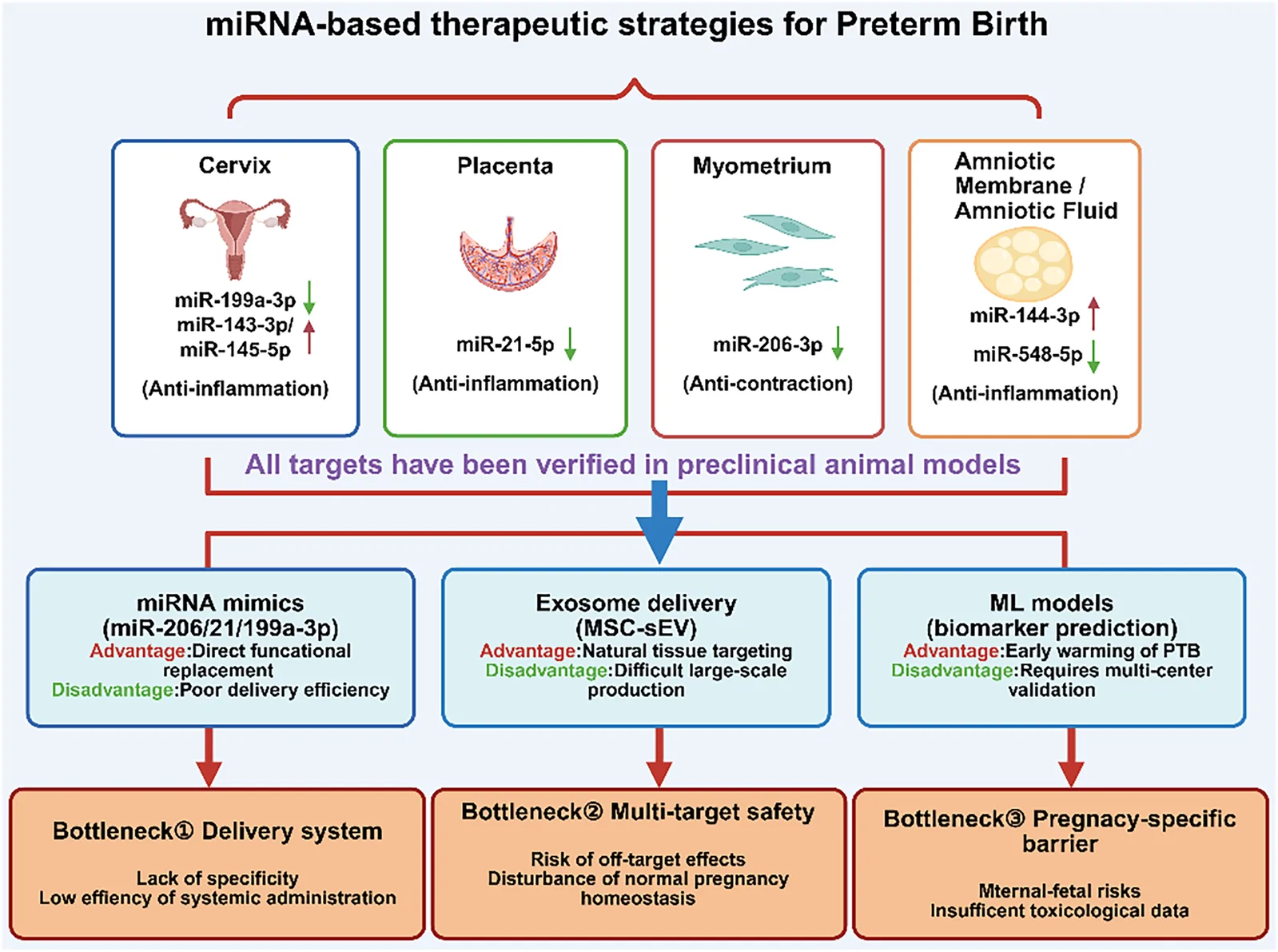
miRNA-based therapeutic strategies for preterm birth: targets, delivery systems, and clinical translation bottlenecks. Four tissue-specific miRNA candidates have been validated in preclinical animal models. Cervix: downregulated miR-199a-3p and upregulated miR-143-3p/145 exert anti-inflammatory effects. Placenta: downregulated miR-21-5p exerts anti-inflammatory effects. Myometrium: downregulated miR-206-5p exerts anti-contraction effects. Amniotic membrane: upregulated miR-144-3p and downregulated miR-548 exert anti-inflammatory effects. Current intervention strategies include miRNA mimics (e.g., miR-206-5p, miR-21-5p, miR-199a-3p), exosome-based delivery (MSC-sEVs), and machine learning models for biomarker prediction. Major clinical translation bottlenecks include: (i) lack of tissue-specific delivery systems; (ii) multi-target safety concerns (off-target effects, disturbance of normal pregnancy homeostasis); and (iii) pregnancy-specific safety barriers (maternal-fetal risks, insufficient toxicological data).

**Table 2:** Summary of miRNA-based therapeutic candidates for preterm birth.

Study	PTB subtype	Target tissue	miRNA/strategy	Mechanism	Target/pathway	Model	Key findings	Development stage	Major limitations	Level of evidence	Biomarker status
Peng (96)	PTB; PPROM	Cervix	miR-199a-3p mimic	Inhibits HMGB1/TLR4/NF-κB inflammatory axis	HMGB1	Preterm patient tissues + *in vitro* cells + LPS-induced preterm mouse model	Reduced HMGB1, IL-6, TNF-α; suppressed cervical inflammation	*In vivo* validation	Delivery efficiency and long-term safety need optimization	observational (human); mechanistic (*in vitro*); animal (*in vivo*)	Discovery-phase biomarker
Anton (97)	Not specified	Cervix	Anti-miR-143-3p/miR-145-5p	Inhibits miR-143-3p-3p-3p/145 to maintain cervical barrier integrity	BCL2, ECM components	Preterm patient tissues + *in vitro* cells	Theoretically helps maintain cervical barrier	Target identification	No experimental validation	observational (human); mechanistic (*in vitro*)	Discovery-phase biomarker
Son (112)	Infection-associated PTB; PPROM	Amniotic membrane	miR-548 mimic	Inhibits HMGB1-mediated amniotic inflammation	HMGB1	Human amniotic epithelial cells (hAECs)	Overexpression suppressed LPS-induced HMGB1 secretion	*In vitro* only	No *in vivo* validation	observational (human); mechanistic (*in vitro*)	Discovery-phase biomarker
Deng (114)	sPTB (animal model)	Myometrium	miR-206-5p agomir	Inhibits gap junction formation, reduces contraction	GJA1 (CX43)	RU486-induced preterm mouse model	Preterm birth rate: 100% → 43%; 4/7 delivered at term	*In vivo* validation	Systemic administration, lack of tissue specificity	animal (*in vivo*); mechanistic (*in vitro*)	Discovery-phase biomarker
Chen (115)	Not specified	Myometrium	miR-26a-5p mimic	Regulates Ca²^+^ signaling, inhibits contraction pathway	TRPC3	Preterm patient tissues + *in vitro* cells	Reduced TRPC3 expression; inhibited CPI17/PKC/PLCγ pathway	*In vitro* only	No *in vivo* validation	observational (human); mechanistic (*in vitro*)	Discovery-phase biomarker
Duan (116)	Infection-associated PTB (animal model)	Placenta	miR-21-5p mimic	Inhibits NF-κB-mediated inflammation	NF-κB p50	LPS-induced infectious preterm mouse model	Suppressed NF-κB, IL-6, TNF-α expression	*In vivo* validation	Only anti-inflammatory effect validated	mechanistic (*in vitro*); animal (*in vivo*)	Discovery-phase biomarker
Wang (118)	PPROM	Chorion	miR-150-5p/ADAM19 axis	Inhibits chorionic cell migration/invasion	ADAM19	Preterm patient blood + *in vitro* cells	Prediction efficiency for PPROM: 94.21%; AUROC 0.8508(small sample size; no external validation; risk of overfitting)	*In vitro* only; Target identification	Primarily a diagnostic biomarker	observational (human); mechanistic (*in vitro*)	Discovery-phase biomarker
Li (123)	sPTB; PPROM	Amniotic membrane	miR-144-3p mimic	Inhibits c-fos/COX2, downregulates PGE2 synthesis	c-fos, COX2, PGE2	Preterm mouse and human amniotic membrane tissues	Overexpression reduced PGE2 secretion	*In vivo* validation	No targeted delivery system for Amniotic membrane	observational (human); mechanistic (*in vitro*); animal (*in vivo*)	Discovery-phase biomarker
Tscherrig (124)	Infection-associated PTB (animal model)	Placenta/fetal brain	MSC-sEV (miRNA cargo)	Exosome-mediated miRNA delivery; promotes oligodendrocyte maturation	TP53, TAOK1	LPS-induced preterm white matter injury model	Reduced white matter injury; promoted myelination	Preclinical exploration	Delivery efficiency and safety need optimization	mechanistic (*in vitro*); animal (*in vivo*)	Discovery-phase biomarker
Ghafourian (125)	Not specified	Multiple tissues	Engineered exosomes	Surface-modified targeting peptides for miRNA delivery	—	—	—	Proof-of-concept	Placenta/cervix-targeting exosome technology immature	mechanistic (*in vitro*)	Discovery-phase biomarker

#### 5.1.5 Additional miRNA-based therapeutic strategies

Beyond the direct use of miRNA mimics or inhibitors, exosome- and stem cell-based miRNA delivery strategies have recently demonstrated preclinical therapeutic potential. Tscherrig et al. found that mesenchymal stem cell-derived small extracellular vesicles (MSC-sEVs) carry multiple miRNAs (including miR-22-3p, miR-21-5p, miR-27b-3p, and the let-7 family) and show promise for the treatment of white matter injury in preterm infants (124, 126). These sEV-encapsulated miRNAs promote oligodendrocyte maturation and reduce neuronal apoptosis by suppressing TP53 and TAOK1 gene expression. Knockdown of DROSHA, a key enzyme in miRNA processing, reduced miRNA expression within sEVs and abolished their protective effects, indicating that miRNAs represent key effector components mediating, at least in part, the therapeutic efficacy of MSC-sEVs (124). This finding provides a theoretical basis for the use of exosomes as miRNA delivery vehicles. A review by Ghafourian et al. systematically summarized the role of exosomes in pregnancy-associated diseases, highlighting the dual potential of exosomal miRNAs as both diagnostic biomarkers and therapeutic delivery vehicles (125). Selvakumar et al. systematically reviewed the preclinical therapeutic potential of lncRNA sponges, miRNA mimics, miRNA inhibitors, and exosome-associated miRNAs in preeclampsia, providing important methodological references for the application of miRNA-based therapies in the field of preterm birth (127). Furthermore, Riley et al. and Swingle et al. respectively discussed the prospects of non-viral RNA delivery systems in pregnancy, noting that the safety of carriers such as lipid nanoparticles and exosomes during gestation has not been adequately validated, representing one of the core bottlenecks constraining clinical translation (128, 129).

### 5.2 Current bottlenecks and challenges

Despite the progress made in the aforementioned studies, the clinical translation of miRNA-targeted therapies for preterm birth faces multiple obstacles. These challenges are not unique to this field but are particularly prominent in the unique physiological context of pregnancy.

First, the issue of tissue specificity in delivery systems. Systemic administration remains the conventional strategy in most preclinical studies; for example, tail vein injection of miR-206-5p agomir effectively reduced the preterm birth rate (114). However, this approach cannot distinguish diseased tissue from healthy organs. Before reaching target tissues (such as the myometrium, cervix, or placenta), miRNA mimics or inhibitors are widely distributed to organs including the liver and kidneys, potentially causing non-specific gene silencing. Although lipid nanoparticles, polymeric carriers, and exosome-based delivery systems have been extensively explored in cancer therapy (114, 130), safety data regarding their use during pregnancy remain extremely limited. A most direct concern is whether delivery carriers can cross the placental barrier and adversely affect fetal development. Currently, no definitive answer exists (131).

Second, safety risks arising from the multi-target nature of miRNAs. A single miRNA can regulate dozens or even hundreds of target genes, rendering this multi-target characteristic both a therapeutic advantage and a potential risk. Taking miR-21-5p as an example, its role in regulating placental inflammation has been confirmed by multiple studies, yet this miRNA also participates in diverse biological processes including cell proliferation and apoptosis. Suppressing inflammatory pathways in the placenta may confer therapeutic benefits, but if it simultaneously interferes with the function of other genes critical for pregnancy maintenance, unintended consequences may arise (120). The maternal–fetal interface during pregnancy exists in a delicate state of immune balance (121), and non-specific modulation of any given miRNA expression could disrupt this equilibrium (122).

Third, the uniquely stringent safety threshold for medication use during pregnancy. Any drug intended for the prevention or treatment of preterm birth must undergo rigorous safety evaluation in the context of pregnancy. Currently, toxicological data on most miRNA delivery systems in pregnant animal models remain extremely limited. Systematic studies on whether the delivery vehicles themselves possess embryotoxicity, placental toxicity, or teratogenic effects are lacking (128, 132). Even LNP-miRNA formulations that have advanced into clinical trials would require re-evaluation of safety parameters for use during pregnancy. Furthermore, the potential impact of miRNA-based drugs on the maternal immune system, coagulation function, and other physiological processes also warrants careful consideration (133, 134).

These bottlenecks are interwoven and collectively constrain the clinical translation of miRNA-based therapies for preterm birth. Finding breakthroughs to overcome these challenges will be a central task for the next phase of research.

### 5.3 Future directions

To address the above bottlenecks, future research should focus on three directions. First, develop pregnancy-specific delivery systems, such as placenta-derived exosomes or trophoblast membrane-coated nanoparticles, to achieve selective enrichment of miRNA drugs in target tissues. Second, systematically evaluate the applicability and long-term safety of existing candidate targets (miR-206-5p, miR-21-5p, miR-199a-3p) across different preterm birth subtypes. Third, liquid biopsy technologies based on maternal peripheral blood exosomal miRNAs hold promise for early identification of high-risk populations for preterm birth, shifting the therapeutic window to critical stages of disease progression. As delivery technologies mature and safety data accumulate, combination intervention strategies **aimed at** multi-tissue miRNA frameworks are expected to make progress within the next 5–10 years. (**Figure 4** and **Table 2**).

## 6 Discussion

This review elucidates putative multi-tissue synergistic signaling landscape of miRNAs in preterm birth from an integrated perspective encompassing four tissues: cervix, placenta, maternal circulation, and amniotic membrane. The core insight of this framework is that the pathological process of preterm birth is not a localized event confined to a single tissue but rather a networked process involving hypothetical cross-organ communication mediated by miRNAs across multiple tissues (135). Numerous valuable narrative and systematic reviews have previously summarized miRNA research in preterm birth, with many focusing on placental miRNA alterations, circulating miRNA biomarkers, or extracellular-vesicle-associated signatures individually. However, most prior reviews tend to emphasize observations within one single tissue or only one research dimension, without sufficiently elaborating inter-tissue communication and synergistic regulatory relationships across cervix, placenta, amniotic compartment, and maternal circulation. Differing from these previous summaries, the present review assembles evidence across four key gestational tissue compartments to construct a putative multi-tissue miRNA framework for PTB pathogenesis. Beyond biomarker summaries, we further connect this network perspective to pre-clinical miRNA-oriented therapeutic attempts and analyze translational bottlenecks for tissue-targeted intervention. This cross-tissue integrative-centered viewpoint forms the major distinctive contribution of this review.

### 6.1 Operational logic of the multi-tissue network

miRNAs from different tissues exhibit functional complementarity. Cervical miRNAs influence the cervical ripening process by regulating extracellular matrix remodeling and barrier function (136). Placenta-specific miRNAs modulate trophoblast cell invasion, angiogenesis, and immune toleran25-ce (137). Maternal circulating miRNAs serve as non-invasive biomarkers (138). Amniotic fluid miRNAs reflect local inflammatory status (139). Notably, exosome-mediated cross-tissue communication represents an important connection mode within this landscape. Studies have found that exosomal NF-κB inhibitors can delay LPS-induced preterm birth and modulate fetal immune cell profiles (140). Placenta-derived exosomes also offer new possibilities for early diagnosis and treatment of pregnancy complications (125). On the temporal dimension, alterations in cervical miRNAs occur during the second trimester, whereas placenta- and amniotic membrane-associated miRNAs predominantly exert their effects during the third trimester. This pattern of functional complementarity and temporal synergy suggests that different miRNA targets or biomarker panels may need to be selected for preterm birth subtypes occurring at different gestational stages. Integrative analyses have demonstrated that the predicted target genes of differentially expressed plasma miRNAs exhibit concordant expression alterations in the placenta, significantly outperforming single-marker approaches (89). The putative multi-tissue miRNA framework for preterm birth pathogenesis is summarized in **Figure 5**, which distinguishes experimentally validated molecular mechanisms from hypothetical cross-tissue crosstalk inferred from indirect evidence.

**Figure 5:**
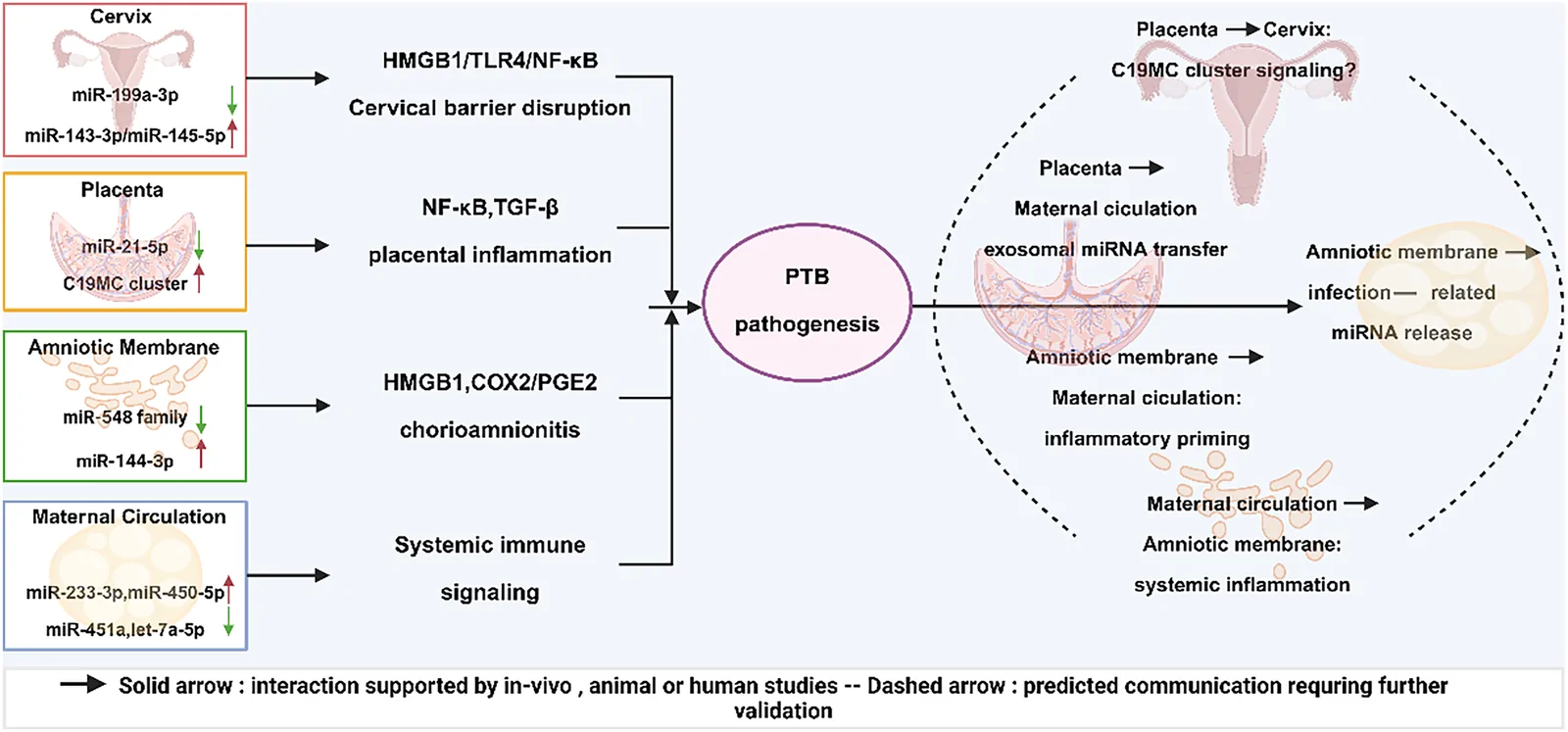
Evidence-hypothesis map of miRNAs in preterm birth. Schematic summary of the multi-tissue miRNA-mediated regulatory framework in preterm birth, integrating four key gestational compartments: cervix, placenta, maternal circulation, and amniotic membrane. The left panel shows experimentally validated mechanisms, in which solid arrows indicate molecular interactions and signaling pathways supported by in-vitro, animal, or human clinical studies. The right panel illustrates hypothetical cross-tissue crosstalk, in which dashed arrows represent predicted communication inferred from indirect evidence. Question marks denote unconfirmed or partially supported regulatory connections that require further experimental validation. Notably, most cross-tissue signaling connections remain hypothetical and await direct experimental confirmation.

### 6.2 Convergent pathways at the mechanistic level

Bioinformatic analyses have identified the NF-κB signaling pathway, TGF-β signaling pathway, progesterone receptor signaling, and extracellular matrix remodeling pathways as core regulatory nodes of preterm birth-associated miRNAs (141). These pathways do not operate in isolation but rather form a dynamically balanced regulatory network. For example, the miR-200 family regulates epithelial–mesenchymal transition and cervical remodeling by modulating ZEB1 and ZEB2 (142). In infection-associated sPTB, miR-223-3p and miR-150-5p exert immunomodulatory functions by regulating cytokine signaling pathways including IL-6ST, TGF-β, and MMP-14, exhibiting negative correlations with pro-inflammatory factors and positive correlations with anti-inflammatory factors (104). This convergence of pathways explains why preterm births of different etiologies can present with similar clinical phenotypes.

### 6.3 Translation from mechanisms to therapy

The aforementioned mechanistic studies not only explain disease pathogenesis but also provide intervention targets. Deng et al. found that miR-206-5p regulates uterine contraction by targeting GJA1, and tail vein injection of miR-206-5p agomir in a RU486-induced preterm birth mouse model reduced the preterm birth rate (114). Duan et al. reported that intraperitoneal injection of miR-21-5p mimic in an LPS-induced infection-associated preterm birth mouse model restored miR-21-5p expression and suppressed NF-κB and downstream inflammatory cytokines (116). Peng et al. demonstrated that miR-199a-3p inhibits the TLR4/NF-κB inflammatory pathway by targeting HMGB1, and *in vivo* transfection with miR-199a-3p mimics in an LPS-induced preterm birth mouse model suppressed cervical inflammation (96). Li et al. reported that miR-144-3p suppresses prostaglandin E2 synthesis by inhibiting c-fos and COX2 expression, and overexpression of miR-144-3p in amniotic membrane tissue reduced inflammation-induced PGE2 secretion (123). These examples illustrate a clear translational chain from mechanistic research to therapeutic development.

### 6.4 Research limitations and future directions

The studies included in this review are predominantly case–control designs, making it difficult to establish causal relationships. Gestational age is an essential confounder in pregnancy biomarker research. miRNA expression fluctuates physiologically with advancing gestation. Some included clinical studies adjusted for gestational age, while many observational and tissue-based studies did not. Lack of gestational age correction may cause biased interpretation of biomarker differences, which may reflect physiological gestational variation rather than pathological changes. Circulating miRNAs cannot be fully distinguished according to their maternal or fetal origins in current studies. The same miRNA may exhibit opposite expression patterns and functional roles across different tissues and preterm birth subtypes, as exemplified by miR-21-5p. The analysis of fetal–maternal bidirectional signaling mechanisms remains insufficient, particularly the routes through which fetus-derived miRNAs, such as the placenta-specific C19MC cluster miRNAs, cross the placental barrier via exosomes to modulate maternal inflammatory responses, which have yet to be elucidated (143, 144). Sample sizes are generally small, and large-scale multicenter validation is lacking. The impact of fetal sex on miRNA expression is often overlooked; differential expression of certain pro-inflammatory miRNAs in placental tissue of male fetuses may explain the sexual dimorphism in preterm birth rates (145). Although the putative multi-tissue miRNA framework provides a novel framework for risk stratification and mechanism-guided intervention in preterm birth, preclinical evaluation and translational research still faces core bottlenecks including the lack of tissue-specific delivery systems and insufficient safety evidence for medication use during pregnancy (129). We acknowledge that the current evidence remains at the discovery stage and that clinical relevance requires further validation. Safety data on carriers such as lipid nanoparticles and exosomes during gestation remain extremely limited, and whether delivery carriers cross the placental barrier or adversely affect fetal development remains unanswered (132). Regarding the safety risks posed by the multi-target nature of miRNAs and the uniquely stringent safety thresholds for medication use during pregnancy, future research requires systematic evaluation (133, 134). With the advancement of single-cell sequencing technologies, it will become possible to dissect the precise roles of miRNAs in specific cell types (146); machine learning-based miRNA prediction models are emerging as breakthrough points in translational medicine, and the integration of multi-omics data with artificial intelligence models has demonstrated superior predictive accuracy compared with single-marker approaches (147, 148). However, clinical standardization still faces challenges including heterogeneity in sample collection protocols (149), cross-ethnic baseline differences in miRNA expression (144), and the lack of FDA-certified detection systems (150). Future efforts should focus on conducting large-scale prospective cohort studies (151) and establishing cross-ethnic, multicenter validation platforms to ultimately achieve a closed translational loop from biomarker discovery to clinical decision support.
